# Nutraceuticals and Food-Grade Lipid Nanoparticles: From Natural Sources to a Circular Bioeconomy Approach

**DOI:** 10.3390/foods11152318

**Published:** 2022-08-03

**Authors:** Cristina Blanco-Llamero, Joel Fonseca, Alessandra Durazzo, Massimo Lucarini, Antonello Santini, Francisco J. Señoráns, Eliana B. Souto

**Affiliations:** 1 Department of Pharmaceutical Technology, Faculty of Pharmacy, University of Porto, Rua de Jorge Viterbo Ferreira, 228, 4050-313 Porto, Portugal; cristina.blanco@uam.es (C.B.-L.); jfonseca@ff.up.pt (J.F.); 2 Healthy Lipids Group, Departmental Section of Food Sciences, Faculty of Sciences, Autonomous University of Madrid, 28049 Madrid, Spain; javier.senorans@uam.es; 3 CREA-Research Centre for Food and Nutrition, Via Ardeatina 546, 00178 Rome, Italy; alessandra.durazzo@crea.gov.it (A.D.); massimo.lucarini@crea.gov.it (M.L.); 4 Department of Pharmacy, University of Napoli Federico II, Via D. Montesano 49, 80131 Napoli, Italy; 5 REQUIMTE/UCIBIO, Faculty of Pharmacy, University of Porto, Rua de Jorge Viterbo Ferreira, 228, 4050-313 Porto, Portugal

**Keywords:** nutraceuticals, lipids, solid lipid nanoparticles, nanostructured lipid carriers, food-grade ingredients

## Abstract

Nutraceuticals have gained increasing attention over the last years due to their potential value as therapeutic compounds formulated from natural sources. For instance, there is a wide range of literature about the cardioprotective properties of omega-3 lipids and the antioxidant value of some phenolic compounds, which are related to antitumoral activity. However, the value of nutraceuticals can be limited by their instability under gastric pH and intestinal fluids, their low solubility and absorption. That is why encapsulation is a crucial step in nutraceutical design. In fact, pharmaceutical nanotechnology improves nutraceutical stability and bioavailability through the design and production of efficient nanoparticles (NPs). Lipid nanoparticles protect the bioactive compounds from light and external damage, including the gastric and intestinal conditions, providing a retarded delivery in the target area and guaranteeing the expected therapeutic effect of the nutraceutical. This review will focus on the key aspects of the encapsulation of bioactive compounds into lipid nanoparticles, exploring the pharmaceutical production methods available for the synthesis of NPs containing nutraceuticals. Moreover, the most common nutraceuticals will be discussed, considering the bioactive compounds, their natural source and the described biological properties.

## 1. Introduction

In recent decades, nanoparticles have increasingly become a subject of interest for researchers, mainly due to the use of materials and production methods that are easy to scale and safe, especially the ones that avoid solvents. These properties make these systems especially attractive for the synthesis of nutraceuticals. A nutraceutical is formed by nutrient compounds, each of which has a pharmaceutical and standardized value, with physiological benefits for human health, performance and well-being, and which is obtained from natural sources. In fact, the description of bioactive compounds includes lowering the risk of developing specific medical diseases, and these compounds include, for instance, bioactive peptides, polyphenols, omega-3 PUFA, probiotics, carotenoids, etc. However, nutraceuticals derived from natural sources are quickly oxidized and unstable, which limits their utilization. On the other hand, the bioactive ingredients present in nutraceuticals are often unable to achieve their potential outcomes due to limited aqueous solubility, leading to a poor bioavailability profile and interaction with gastro-intestinal fluids. Therefore, their encapsulation in order to increase their bioavailability and adsorption is instrumental to improve their therapeutic potential. Recent progress in the field of nutraceutical delivery has incorporated nanotechnology to overcome the drawbacks accompanying nutraceuticals. Nano-based carrier systems provide several benefits, including an undesirable taste, odor and color masking, providing a pH-triggered controlled release, improved stability, improved shelf life, preservation of volatile ingredients, protection against gastric conditions and pH before reaching the target and protection for the ingredients from different environmental parameters, including oxygen, heat, water and light.

Nanotechnology can improve the bioavailability of nutraceuticals by their encapsulation using nanocarriers for proper delivery to their target, or by the transformation of these compounds into a nanoparticle form. This comprehensive review focuses on issues associated with nutraceuticals and nano-scale formulation approaches, describing recent nanodelivery systems used to encapsulate different nutraceuticals or bioactive compounds with biological value and therapeutic properties. In addition, the advantages and disadvantages of the most common encapsulation techniques and nanodelivery systems used to encapsulate nutraceuticals will be explored, taking into account the main challenges related to their stability.

## 2. Sources of Nutraceuticals with Biological Value

Bioactive compounds obtained from natural sources are a widespread and heterogeneous group with highly different biological properties described to be potential alternative therapeutic tools useful in both the prevention or treatment of some diseases. Their therapeutic value has already been proven effective by numerous clinical trials. Most common bioactive compounds can be classified as bioactive lipids (omega-3 fatty acids, oleic acid), carotenoids, bioactive peptides and phenolic compounds, among others [1,2]. Table 1 shows the different more common bioactive compounds, including their natural sources and their proven biological value.

Omega-3 fatty acids are long-chain polyunsaturated fatty acids, and their main bioactive forms are: docosahexaenoic acid, DHA (22:6 Ω-3), eicosapentaenoic acid, EPA (20:5 Ω-3) and α-linolenic acid, ALA (18:3 Ω-3). In fact, ALA is the precursor of the bioactive fatty acids EPA and DHA; nevertheless, its conversion rate in the human body has been described as low; thus, the bioactive forms of EPA and DHA must be obtained from natural sources. The traditional sources of omega-3 fatty acids, including EPA and DHA, have been fish and krill oil. This approach, however, supports the overexploiting of the ocean and abusive fishing. Therefore, new alternative biomasses are needed to obtain EPA and DHA. Microalgae are a promising source of bioactive compounds, especially omega-3 fatty acids, as they are the only nonanimal source of the bioactive form of omega-3. One of the main advantages of microalgae as omega-3 producers is their ability to grow in wastewater without competing with terrestrial plants for arable lands. Microalgae have a grow rate 2–3 times higher than that of terrestrial plants, and they are able to accumulate a wide range of bioactive compounds in their cells depending on the microalgae species. Among them, *Nannochloropsis gaditana*, *Isochrysis galbana*, *Tetraselmis chuii* and *suecia* and *Phaedodactylum tricornutum* are the main species producing EPA and DHA [1,3,4,45,46,47,48]. Omega-3 lipids have been proven to be related to the prevention and treatment of some world-recognized diseases, such as cardiometabolic disease and age-related macular degeneration, with promising results [5,6,7,8,9,10,11,49].

Carotenoids are a widespread group constituted by more than 400 different types of substances, which have been described in natural sources such as vegetables, egg, fish, algae and microalgae. The most common carotenoids included in nutraceuticals are lutein, zeaxanthin, fucoxanthin, astaxanthin and beta-carotene, which have been proven to be related to different biological values in clinical trials [20,21,32,33,34]. For instance, lutein and zeaxanthin have been shown to be alternative and effective treatments for early AMD after supplementation, and they have been proven to influence neurological and visual development during pregnancy. Lycopene has been shown to be able to reduce the risk of prostate cancer due to its antioxidant activity and ability to realize the induction of the apoptosis, the inhibition of cellular growth, the decrease in IGF-1 and IGF-BP-3, the induction of phase II enzymes and the modulation of androgenic metabolism [2,20,21,22,23,24,33,50,51,52].

It is interesting to highlight the wide biological properties that this group of bioactive peptides shows depending on the peptide studied, including antihypertensive, antioxidant, antiproliferative, anti-inflammatory, apiaceous, hypocholesteremia, antithrombotic and mineral chelator functions [40,41,42,43,44,53]. On the other hand, phenolic compounds, including flavonoids (anthocyanins, flavanols, catechins, gallo catechins, etc.), phenolic acids (caffeic acid, vanillin acid, etc.), lignans and stilbenes, show mainly antioxidant and antitumoral activities related to the reduced risk of cardiovascular disease due to the inhibition of LDL oxidation, an antihypertensive effect, anti-inflammatory effect and regulation of the immune response, platelet antiaggregant. Isoflavones found in soy, on the other hand, are associated with an antiestrogenic effect due to their interaction with 17-b-estradiol receptors [35,36,37,38,39,54,55,56].

## 3. Challenges Encountered in Nutraceutical Stability

Due to the complex nature of the different available bioactive compounds and the heterogenicity of them all (including molecular weight, charge, thermosensitivity or polarity), there are important challenges related to the properties of each one that must be considered. First of all, the low solubility of most of the bioactive compounds described should be highlighted, including, for instance, bioactive lipids or carotenoids. Due to this fact, optimal formulations must be designed to encapsulate these compounds. Indeed, if the final purpose of using a nutraceutical is its inclusion in the food matrix, the dosage of the bioactives is crucial, whereas micro/nanoemulsions must be produced to make them dispersible in the often-aqueous food matrix and resistant enough to pass through the rest of the food process. Additionally, the nutraceutical must be delivered in the target area at a controlled velocity, which will depend on the nanoparticle’s constituents. Regarding bioactive lipids, their low solubility, the crystallinity of some at room temperature and their thermosensitivity, which forbids the use of high temperatures in the process, should be highlighted. Additionally, the formulation should protect the bioactive from the stomach pH and digestion, maintaining the biological properties after these processes. This is also a crucial point for bioactive peptides, which should be protected from high temperatures, physiology pH and organic solvents, which may damage their structure and make them lose their biological value. Additionally, as some of them are bitter, they benefit from an adequate vehiculation system masking their flavor. Regarding phenolic compounds and phytochemicals, their solubility is affected by their polarity, functional groups, molecular weight, if it is oxidized or reduced, or if it is complexed to another molecule. Therefore, each compound should be studied deeply before choosing one production technique or another to preserve their biological value and to load it efficiently [57,58,59,60,61,62].

**Figure 1:**
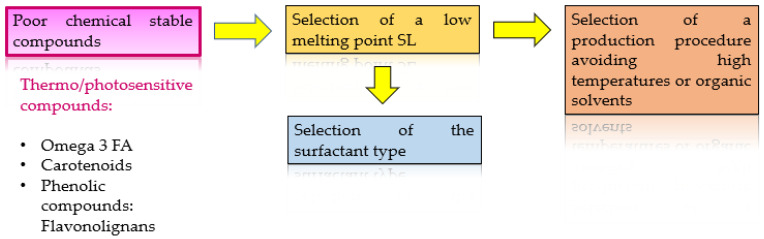
Diagram representing the key points to consider in nanoparticle formulation for loading unstable bioactive compounds.

Taking all these aspects into account, the main key points that must be considered in the formulation of NPs including nutraceuticals are shown below (Figure 1).

The main principles of different nutraceuticals to be considered in the selection of wall materials and nanodelivery system production procedures are described below:

- Bioactive solubility: the selection of wall lipid materials relies on physiological tolerance, physiochemical structure, active principle’ solubility and solid lipid–liquid lipid miscibility. Solubility of bioactive compounds in the lipidic matrix is one of the most important factors determining its loading capacity. Preliminary studies must be performed to study this factor with each compound and each solubility, taking into account their polarity, functional groups, molecular weight, if it is oxidized or reduced or if it is complexed to another molecule. Indeed, most of the bioactive compounds have low water solubility, including carotenoids, bioactive lipids and some phenolic compounds, whereas bioactive peptides are usually more hydrophilic [42,43,44].
- Wall materials’ compatibility: in NLCs, liquid lipid and solid lipid molecules should have good miscibility and compatibility with each other. This prevents the formation of the solid lipid crystalline matrix, promoting an amorphous structure typically presented in NLCs. The ideal ratio between liquid lipids and solid lipids is reported to range from 70:30 up to a ratio of 99.9:0.1 [63].
- Bioactive stability: most of the bioactive compounds described are highly unstable during oxidation, including carotenoids, lipids and phenolic compounds. The lipid matrix plays a crucial role in the protection of the bioactive ingredients. Wall materials should be stable against chemical degradation, including oxidation and lipolysis. Medium-chain triglycerides (MCTs) are the most common oils used in NP production. They have a small molecular weight and are water-soluble. Moreover, MCTs’ (e.g., Miglyol 812) digestion is faster than that of long-chain triglycerides (e.g., corn oil), and they have higher stability against oxidation. Additionally, they are generally recognized as safe (GRAS) by the US Food and Drug Administration (FDA) for direct addition into many foods, including beverages, as a carrier, solvent and emulsifier. Oleic acid or PUFAs are commonly used in the food and pharmaceutical industries. However, their susceptibility to oxidation may cause damage to encapsulated compounds through production of free radicals; thus, their use should be limited to highly stable compounds, avoiding carotenoids or omega-3 lipids. Emulsifiers have been used for stabilization of the lipid dispersions by reduction in interfacial tension between the lipid phase and the aqueous phase during the production of the particles, leading to fine nanocarriers. It has been found that utilization of a mixture of emulsifiers can be more efficient in preventing particle aggregation. A combination of tween 80 and lecithin caused smaller particles with a lower PI and higher stability due to an increased zeta potential in comparison to their use separately. Additionally, compared to ionic surfactants, tween 80 has low toxicity and is approved for use in specific food products and is GRAS [64,65].
- Bioactive thermosensitivity: the method used for NP production should be selected based on the bioactive compound properties, thermosensitivity and water solubility, which are the main factors affecting its selection. Homogenization at high pressure is the most used method in food industry; however, the high temperatures employed in this technique have led to the development of cold homogenization, which could be more adequate for carotenoids, bioactive lipids and peptides [65,66,67].

## 4. Nanoparticles Used for the Loading of Nutraceuticals: Food-Grade Nanosystems

Overcoming the main drawbacks related to some bioactive compounds, such as low water solubility, poor chemical and oxidation stability associated with thermosensitivity and photosensitive compounds, gastric degradation and thus poor bioavailability, must be the focus. Many of these challenges, such as the low water solubility, gastric degradation and the poor bioavailability, can be overcome by encapsulating these compounds into nanoparticles as efficient delivery vehicles contributing to the industry’s economy. A circular bioeconomy has become a model for commercial production that enhances reuse, recycle and recovery with a smaller environmental footprint in nutraceutical industries. Nutraceutical industry costs are mainly related to the challenges mentioned above, requiring large numbers of bioactive ingredients to achieve an adequate in vivo effect, so many natural biomasses are still unexploited [68,69]. Nutraceuticals’ production using lipid nanoparticles as carriers allows for their obtention in one stage of production with higher encapsulation efficiencies, as was reported in previous works, providing economic advantages, decreasing the number of process steps and the dosage of nutrients by increasing their bioavailability [66,70,71,72,73]. NPs overcoming the main challenges related to nutraceuticals have been widely reported in the scientific literature. Recent works on NP nutraceutical release showed an increased water solubility and in vitro bioavailability of curcumin [72]. Improved oxidative stability has also been described in NP omega-3 works [74]. Thus, the encapsulation of bioactive compounds in nanocarriers allows for the incorporation of natural biomasses into the circular bioeconomy.

Interestingly, lipid NPs’ structure can overcome the abovementioned poor bioavailability by different causing factors. The main critical steps in the oral absorption of nutrients could be the rate of dissolution and the rate of nutrient penetration across the bio membrane. An essential prerequisite for the absorption of a bioactive compound is its ability to exist in a stable aqueous solution. This fact depends on its aqueous solubility and dissolution rate. Lipid-based nanoparticles improve the bioavailability due to their increased surface area. Additionally, by decreasing the particle size, the thickness of the diffusion layer is decreased, leading to faster transport and faster dissolution [57,63,64].

Going deeper, lipid NPs promote enhanced gastrointestinal (GI) absorption due to induced permeability changes caused by the surfactant, and due to the increased residence time in the stomach and upper small intestine owing to their lipidic nature and their adhesion to the intestinal underlying epithelium. The bioactive compound is protected from the harsh gastric conditions as it is encapsulated in the NPs, promoting their stability. On the other hand, NPs have low stability in acidic environments, which make them degradable by gastric lipases [75]. Thus, lipid NPs are transformed by lipase and colipase into micelles (consisting of bioactive and lipid monoglycerides), stimulating bile flow to form mixed micelles. Mixed micelles are absorbed by chylomicron formation into lymphatic vessels, avoiding the first-pass effect and enhancing drug bioavailability alongside the fat absorption process. Through systemic and lymphatic transport, they increase the concentration of nutrients in the systemic circulation. On the other hand, nanoparticulate systems were reported to improve oral drug bioavailability by intracellular uptake by M cells of Peyer’s patches. Indeed, transient opening of tight junctions (gaps between two adjacent intestinal epithelial cells) has been reported due to the effect of highly lipophilic surfactants, improving paracellular absorption [63].

Moreover, bioactive compound release from lipid particles occurs by diffusion and simultaneous degradation of lipid particles in the body. Controlled release from NPs can lead to a prolonged half-life and slows down the enzymatic attack in systematic circulation. The degradation rate, and therefore the kinetics of compound release, depend on the type of lipid used. The shorter the fatty acids of triacylglycerols, the faster the degradation rate. Surfactants including lecithin or sodium cholate can play an important role in accelerating the degradation rate by inducing attachment of the lipase/colipase to the nanoparticle [65,76].

Other main drawbacks must be studied to achieve an adequate formulation. Therefore, in order to produce lipid NPs with thermosensitive compounds, including omega-3 FA, carotenoids and flavonolignans, the selection of a low-melting-point lipid, such as glyceryl monooleate, glycerin, Monosteol™ or Softisan™, and a production procedure avoiding high temperatures, is a crucial step. It is also important to avoid the use of organic solvents that could damage the stability of the bioactive compounds, and to work with photosensitive compounds, opaque lipids must be selected as formulation components. Lipid components, solids and liquids (in the case of NLCs), will be selected based on these points, and based on their miscibility with the bioactive compounds.

Once the lipid components are selected, the surfactant type should be chosen as a function of the former. The lipid and surfactant content in the formulation will be the main factors affecting the chemical properties of the nanoparticles developed. Higher numbers of solid lipids (SL) will lead to a larger particle size, although it will also depend on the surfactant content [70,77]. However, to increase the NPs’ load, it is necessary to increase the SL rate, and thus, compromise conditions must be selected for each case. Frequently, lipid NPs are produced through the combined use of Ultra-Turrax with sonication to reduce particle size. Nevertheless, the addition of the sonication step can also increase the polydispersity index and particle size distribution. On the other hand, when the target administration is intravenous or ocular, the rheology of the NPs produced must be studied to explore the NPs’ behavior under different conditions. Ideally, the viscosity must decrease with the stress applied, whereas the loss and modulus storage must increase, with the storage always being higher than the loss (G′ > G″), to achieve an appropriate rheological behavior upon administration [25,58,59,60,62,70,77,78,79,80,81,82,83,84,85]. The lipid-based nanosystems solid lipid nanoparticles (SLNs) and nanostructured lipid carriers (NLCs) will be described, as well as the component types and the production procedures available. Table 2 lists the latest works on the production of NPs encapsulating nutraceuticals, highlighting the ones focused on lipid nanoparticles. Recent studies have also investigated the use of resistant starch or soy-protein-based polymeric nanoparticles for the encapsulation of ferulic acid and curcumin, respectively, showing higher stability of the nutraceutical loaded [60,84]. 

Lipid nanoparticles are similar to polymeric ones in terms of the solid matrix structure that they share. However, lipid nanoparticles appear to avoid the problems of toxicity of some polymers and solvents used in their production. Lipid nanoparticles are based on biodegradable lipids and emulsifiers, either lipophilic or hydrophilic. Among lipids, those mostly used were tristearin and tripalmitin, whereas amongst the surfactant agents, soy lecithin and polysorbate 80 should be emphasized as lipophilic agents and poloxamer 188 and tween 80 as hydrophilic ones [31,54,70,80,81,82,83].

The other advantage of these kinds of systems is their ability to encapsulate either lipophilic or hydrophilic molecules, and the possibility of covering them with polymers to modify their characteristics, such as the use of polyethylene glycol or chitosan to modulate the permanence of the molecules in the mucous membranes of the organism. Indeed, lipid nanoparticles are versatile systems that can be administrated by different pathways, such as intravenous, oral, cutaneous, pulmonary, ocular and transdermal pathways. They also have limitations though, since during storage, the purest lipids tend to crystalize in a perfect crystal structure that may lead to the drug or bioactive being loaded. That is why a new generation of lipid nanoparticles appeared to solve this problem, the nanolipid carriers (NLCs), whose structure is just imperfect enough to avoid the expulsion of the loaded drug, which is achieved by formulations including not only solid lipids but liquid and solid lipids in combination, which increases the encapsulation efficiency and minimizes the expulsion of the bioactive compounds inside during the storage [25,58,59,60,62,70,77,78,79,80,81,82,83,84,85,86].

## 5. Production Procedures

The major objective of the encapsulation is creating coating-sensitive compounds or reducing side effects of some useful compounds applied in high concentrations; these compounds are located in the core and coated by suitable wall materials. Encapsulation techniques protect nutraceuticals or bioactive compounds from unbalanced and unfavorable conditions, including pH, light, moisture, heat, chemical and biological degradation, and oxygen during storage, processing and utilization. Wall materials, including lipids and surfactants, have a critical role in the encapsulation technique because of their important effects on target delivery, bioavailability, biocompatibility and protection of bioactive compounds. Additionally, these materials should be safe and do not have an impact on flavor, color, texture or other properties of foods. The most important properties of suitable wall materials include a low cost, low viscosity, film-forming capacity, high solubility, low hydroscope, high stability in the media of the target, high protection, abundance, nontoxicity and compatibility in food or drug formulations. Several techniques are used for encapsulating bioactive agents; the preferred encapsulation technique depends on the bioactive compound structure and its end use. The most common encapsulation techniques for lipid NPs include emulsification, homogenization at high pressure, microemulsion and emulsion–evaporation of the solvent, sometimes combined with sonication. The main production procedures are discussed below for their use in the food industry.

### 5.1. Homogenization at High Pressure (HPH)

The melted lipid is emulsified in an aqueous solution containing the surfactant at the same temperature by agitation at high speed or ultrasounds. The pre-emulsion is then subjected to high-pressure homogenization. As typical production conditions, 500 bar pressure and between 3 and 5 homogenization cycles are repeated. Finally, the nanoemulsion is cooled, the lipid phase solidifies, and the suspension of lipid nanoparticles is formed. It must be highlighted that increasing homogenization cycles may lead to particle coalescence, resulting in a bigger particle size. This technique is especially aimed at the encapsulation of lipophilic molecules since the hydrophilic ones diffuse in a large proportion to the aqueous phase during the homogenization phase, giving rise to a low encapsulation efficiency. One of the drawbacks of this technology is the exposure of the active ingredients to high temperatures, although for a very short time, this allows sensitive compounds to resist the process. Additionally, the high temperatures used in hot HPH may reduce the emulsifying capacity of most surfactants, therefore causing nanocarriers’ instability [64,66,67,74].

For the encapsulation of thermosensitive compounds, a cold homogenization method was designed in which the molten lipid is rapidly cooled in dry ice, the solid form of carbon dioxide, or in liquid nitrogen. In this way, the fragility of the lipid is increased to facilitate the grinding process for obtaining microparticles. These are dispersed in the cold solution of the surfactant, and finally, the suspension is subjected to high-pressure homogenization at or below room temperature [87]. HPH is the most used production technique for nanocarriers encapsulating food ingredients due to the advantages that it has compared to other methods, including large-scale production, disuse of organic solvents and shorter production time.

### 5.2. Preparation Technique via Microemulsion

This method requires low energy and is based on the basic mechanism of microemulsions, which can be transformed into an ultrafine nanoemulsion after their rupture by adding a certain volume of water.

In the microemulsion formation, the lipid melts and the active substance or a drug is dissolved in it. Next, the surfactant, cosurfactant and water are added at a high temperature to form the microemulsion, which is poured over cold water, breaking into nanoparticles of emulsion, which crystallize to form lipid nanoparticles. As drawbacks of this process, we can point out the high concentration of the surfactant and cosurfactant which is required, the use of solvents to form the emulsion and the high dilution to which the particles are subjected, which leads to the final content in particles being below 1%. The temperature difference between the chilled water and the microemulsion extremely influences the particle size in this method. The faster the solidification, the smaller the particle sizes. Although this method is operated under mild conditions, it requires abundant surfactant and cosurfactant, which could be a disadvantage for its use in the food industry [63].

### 5.3. Solvent Emulsification–Evaporation Technique

In this method, very low or no energy is required, and it is widely used for the preparation of polymeric micro- and nanoparticles. The lipid material in this case is dissolved in a water-immiscible organic solvent, in which the active ingredient is also dissolved. This organic phase is emulsified with the aqueous phase containing the surfactant agent by means of mechanical agitation or an ultrasound probe. After evaporation of the solvent at reduced pressure, the dispersion of nanoparticles occurs after the precipitation of the lipid. The preparation of double emulsion in this technique allows the encapsulation of numerous compounds. As there is no heat involved, this method is suitable for heat-sensitive active compounds. The low energy required is another great advantage of this method. The main disadvantages of this technique are solvent-residue-associated toxicity and diluted particles. These disadvantages can be reduced by the selection of a food-grade solvent such as ethanol or ethyl acetate, making this method a good option for the encapsulation of food ingredients [63,77].

### 5.4. Solvent Emulsification–Diffusion Technique

This technique is similar to the previous one, differing only in the method of precipitation of the lipid from the emulsion. In this case, it is achieved by adding extra water to the aqueous phase, which causes immediate diffusion of the organic solvent, with the consequent precipitation of the lipid.

In the solvent emulsification–evaporation process, the lipid is dissolved in the water-immiscible solvent, and then it is emulsified in an aqueous phase containing the surfactant, followed by evaporation of the solvent under reduced pressure. Lipid precipitation occurs upon solvent evaporation, leading to nanocarriers’ formation. Merits of this method include its lab-scale acceptability, higher stability and ability to obtain the smallest particle size, but its demerits are the use of toxic solvents, the increase in lipid content, which leads to an increase in the polydispersity index, and particle size distribution [31,78].

## 6. Conclusions and Future Prospects

Lipid nanoparticles are especially interesting for oral administration, for different reasons, including, in the first place, the mucoadhesive properties that they present due to their colloidal nature, and to which their ability to facilitate the release in the area of the intestine to which they adhere is attributed. On the other hand, there is the possibility that they are internalized by the intestinal cells, and the promoting effect of the absorption of the constituent lipid components must also be considered. Nanotechnology has wide applications in nutrition, food supplements, nutraceuticals and medical science [88,89,90]. The recent literature suggests that nanotechnology will overcome the current main challenges that bioactive compounds and nutraceuticals must face, such as their stability, low solubility, targeted delivery and prolonged release. Additionally, with regard to the food industry, new products must avoid problems related to their color, flavor or nutrient content. Accommodation of each health need could be achieved with the aid of pharmaceutical nanotechnology. In fact, it seems like a promising technology approach to reduce the dose levels and to achieve better and longer stability of the nutraceuticals. The formulations of the bioactives as nanostructured products will help in their superior characterization, improved patient acceptability and, above all, high reproducibility of their therapeutic effectiveness. Thus, a lot of nutraceuticals in nanosized forms have been developed in many works regarding the optimum production procedure or the most adequate wall materials for each nanoparticle, considering lipid types and surfactants. Additionally, many nutraceutical products containing NPs are commercially available on the market. Therefore, it can be concluded that nano-based carrier systems provide better means for enhancing the efficacy and availability of nutraceuticals having issues with solubility, stability and bioavailability.

Nevertheless, components of lipid NPs should be carefully selected since they will directly influence product stability and effectiveness. For future prospects, it should be remarked that studies on orally administered NPs are still very limited, and the molecular mechanisms by which they are absorbed through the intestinal lumen into the circulation should be better clarified by studying each lipid component. Although NPs possess great potential as delivery carriers, more preclinical and clinical studies are needed to better understand their behavior. Additionally, NPs have some related challenges, such as the need to improve their colloidal stability under harsh conditions, including food processing (heating, high pressure, drying, etc.) and the gastrointestinal environment (low pH, bile salt and digestive enzymes); studying interactions between bioactive compounds and nanoparticles for optimal encapsulation; and accepting the biological fate of these nanoparticles upon oral administration. Thus, further investigation on food nanotechnology is needed with regard to the in vivo and food processing stages.

## Figures and Tables

**Table 1:** Source of common nutraceuticals and biological properties, including bioactive lipids, carotenoids, phenolic compounds and bioactive peptides.

Bioactive Compound	Sources	Biological Value	References
Lipids			
Omega-3 EPA and DHA	Fish, krill, microalgae including *Nannochloropsis gaditana*, *Isochrysis galbana*, *Tetraselmis chuii* and *Phaedodactylum tricornutum*	Anti-inflammatory properties Antitumoral effect on in vitro studies on breast, prostate and colon cancer Action during nursing and pregnancy associated with tissue growth, visual and neuronal function development at a dosage of 450 mg of DHA and EPA per day Cardiovascular effect due to the inhibition of the atheroma plaque formation, prevention of arrythmias and antithrombotic effect at a dosage of 250 mg per day High daily dosage of EPA and DHA (6 g) are associated with a low risk of AMD and neurological disease such as TDAH or depression Evidence for the treatment of autoimmune diseases such as rheumatoid arthritis and psoriasis, as well as inflammatory intestinal diseases such as Crohn’s disease or ulcerative colitis	[1,3,4,5,6,7,8,9,10,11,12,13,14]
Oleic acid (18:1 Ω -9)	Olive oil	Prevention and treatment of cardiovascular disease, lowering the blood pressure and the synthesis of TXB_2_	[15,16,17,18,19]
Conjugated linoleic acid (CLA)	Beef, milk, lamb	Increases β oxidation of fatty acids Modulates the release of adipokines and cytokines Modulates the metabolism of adipocytes Increases spending of energy	[17,18]
Carotenoids			
Lutein and zeaxanthin	Yolk egg, marigold flower, green vegetables, especially spinach, fruits and microalgae including *Chlorella vulgaris*, *Scenedesmus almeriensis* and *Nannochloropsis gaditana*	Antioxidant properties Lutein and zeaxanthin are mainly associated with retinal and neurological health at a dosage from 14 to 40 mg per day Reduction in vision loss in AMD patients after supplementation Retinal protection from light exposure and UV radiation, reduction in the associated oxidative stress	[1,2,8,9,20,21,22,23,24,25,26,27,28,29]
Astaxanthin	Crustaceous, fish including salmon and microalgae including *Haematococcus pluvialis*	10 times more antioxidant than b-carotene	[20,21,23,30]
b-carotene (pro-vitamin A)	Carrots, microalgae including *Dunaliella salina*	Vision function Antioxidant	[1,2,8,20,21,23,31]
Lycopene	Tomato and derivates	Is the dietary carotenoid with the strongest antioxidant effect Association with lower risk of prostate cancer due to its antioxidant activity, the induction of the apoptosis, the inhibition of the cellular growth, decrease in IGF-1 and IGF-BP-3, induction of phase II enzymes, modulation of androgenic metabolism	[20]
Fucoxanthin	Brown algae and microalgae including *Phaedodactylum tricornutum*	Antidiabetic and antiobesity properties due to the stimulation of lipolysis and inhibition of lipogenesis, increase in b-oxidation of FA, inhibition of adipocytes differentiation in murine models	[1,20,21,23,32,33,34]
Phenolic compounds			
Flavonoids: anthocyanins, flavanols, catechins, gallocatechins Phenolic acids: caffeic acid, vanillin acid Lignans Stilbenes: Resveratrol	Fruits (grapes, red fruits, citric fruits), vegetables (soy, rosemary, salvia) coffee, tea, cocoa, olive oil	All phenolic compounds present antioxidant, anti-inflammatory and antitumoral effects The antioxidant properties of the phenolic compounds, including catechin and quercetin, are associated with a reduced risk of cardiovascular disease due to the inhibition of LDL oxidation, the antihypertensive effect, anti-inflammatory effect and regulation of the immune response, platelet antiaggregant Resveratrol is associated with an anticancerogenic effect in the prevention and treatment due to the induction of the apoptosis of damaged cells, inhibition of angiogenesis in the tumoral tissues Isoflavones found in soy are associated with an antiestrogenic effect due to their interaction with 17-b-estradiol receptors Curcumin is reported to be a potent anti-inflammatory agent	[35,36,37,38,39]
Protein compounds			
Bioactive peptides	Milk, soy, meat, eggs, algae, fish, wine, cereals	Antihypertensive, antithrombotic, antioxidant, antiproliferative, anti-inflammatory, apiaceous, hypocholesterolemic, antithrombotic, mineral fixative effects Inhibition of ECA: NWGPLV (soy), LKP, IKP, LRP (fish), IKW, LKW (meat), lactochinins and casoquinine (milk), ookinin, KVREGTTY (egg) Immunomodulation: IAP, immunopeptides (wheat), YPK (broccoli), GYPMYPLR (rice) and TTMPLW (milk) Opioids: exorphins A4, A5, B4, B5, C (wheat), casomorphins, lactoferroxin, casoxins (milk) Antimicrobials: f 109–200 (egg), lactoferricin (milk) Antithrombotic: K-CN, casoplatelins (milk) Chelator of metals: casein phosphopeptides (milk) Hypocholesterolemic: LPYPR (soy), IIAEK (milk) Antioxidants: MY (fish), MHIRL, YVEEL, WYSLAMAASDI (milk)	[40,41,42,43,44]

**Table 2:** Overview of the recent works on nanoparticles containing nutraceuticals: Type of nanoparticles (NPs), NP components, bioactive compound encapsulated and production procedure employed are detailed.

NPs	NPs Component	Nutraceutical	Production Procedure	Major Findings	References
NLC, SLN, LNE	Imwitor 900 K, medium-chain triglyceride (MCT) Lipoid^®^ SPC-3, pure soybean phosphatidylcholine, Tween^®^ 80 (polysorbate 80) and Span 20	Quercetin	HPH	Maximum bioaccessibility was observed with LNE compared to SLN and NLC Bioaccessibility is affected by lipid’s physical state and composition	[70]
NLC	Palmitic acid (PA), polyoxyethylene sorbitan monolaurate (Tween 20), ethanol and acetone	B-carotene	Solvent diffusion	Lipid phase and surfactant concentrations have an important effect on particle size Liquid lipid content in NLCs and temperature significantly affect β-carotene degradation	[31]
NLC	Fish oil (FO) with omega-3 fatty acid composition, carnauba wax (CW), glycerol stearate (GS), Poloxamer 407 Tween 80	Lutein	Melting emulsification coupled with the high-shear homogenization technique	Fish oil concentration was found to enhance the lutein entrapment efficiency In vitro lutein release from lipid nanocarriers is slower than that from nanoemulsion.	[25]
NLC	2.7% Q, 9.4% soy lecithin, 23.6% glyceryl tridecanoate, 6.7% glyceryl tripalmitate, 13.4% vitamin E acetate, 44.2% Kolliphor HS15 and an aqueous mixture containing 1% of NaCl in deionized water	Quercetin	Phase-inversion-based process	Q-NLCs decrease the viability of breast cancer cells and induce their apoptosis Q-NLCs increase cellular uptake of Q by breast cancer cells Q-NLCs enhance solubility and stability of Q in aqueous solution	[54]
SLN	Compritol 888 ATO, Pluronic F68 and 1,7-octadiene	Curcumin	High-shear homogenization and ultrasonication techniques	Solid lipid nanoparticles were stable for over 1 month at 4 ± 1 °C In vitro studies showed a good release of curcumin from lipid nanoparticles Formulation increases the amount of curcumin permeated by 2 orders of magnitude	[72]
SLN, NLC	glycerol monostearate (GMS), soy lecithin, Tween 80, glycerol distearate (GDS) (Precirol Ato 5), medium-chain triglyceride (MCT) (Labrafac Lipopile WL 1349)	Zeaxanthin	Homogenization at high speed and ultrasonication	Encapsulation efficiency and loading are higher for NLCs than SLNs	[85]
SLN	Witepsol H15 and Polysorbate 80 (Tween 80)	Rosmarinic acid	Hot-melt ultrasonication method	High association of rosmarinic acid was detected, and stable particles were obtained	[58]

## Data Availability

Not applicable.
